# Acute and Complete Expulsion of a Porous Polyethylene Orbital Implant 14 Months after Primary Enucleation for Uveal Melanoma: A Case Report

**DOI:** 10.1055/a-2534-2276

**Published:** 2025-04-16

**Authors:** Julien Schaller, Augustina Grigaité, Andréas J. Kreis, Aurélie Oberic, Barbara Spahn, Ann Schalenbourg

**Affiliations:** 1 Ophthalmology, Hôpital ophtalmique Jules-Gonin, Lausanne, Switzerland; 2 Ophthalmology, HUG, Geneve, Switzerland; 3 Ophthalmology, Ophthalmology Clinic, Lausanne, Switzerland

## Introduction

The removal of the eye, known as “enucleation,” is still a commonly used treatment strategy for intraocular malignancy such as uveal melanoma or retinoblastoma, the other major indications being severe trauma and a blind painful eye 1 , 2 . To obtain optimal cosmesis and comfort, a “double” replacement technique is currently employed. During surgery, a spherical orbital implant is introduced into the anophthalmic socket, onto which the recti muscles are sutured to both replace the eye volume and maintain its motility 3 . Six to eight weeks later, once the conjunctival wound is healed, a cosmetic prosthesis, the “artificial eye,” with the shape of a shell made from glass or resin, is adapted, and placed in the conjunctival sac.

Historically, orbital implants have been used since 1885. Several materials have been experimented with, such as glass, cartilage, fat, bone, silk, wool, aluminum, cork, ivory, Vaseline, paraffin, etc. The newer generations of orbital implants are either “nonporous,” made from acrylic or silicone, or, more recently, “porous,” made from hydroxyapatite (HA) or polyethylene (PP). The advantage of the latter is that they allow fibrovascular tissue ingrowth, leading to bio-integration of the implant within the hostʼs tissue, and thus lowering the complication rate of infections as well as implant migrations and extrusions 2 , 4 , 5 , 6 , 7 , 8 , 9 .

PP is a high-density straight-chain hydrocarbon material, formed by polymerization of ethylene molecules. It is reported to be nontoxic, nonallergenic, and biocompatible and the material has been widely used for nose, ear, and orbital reconstruction 2 . Medpor (Stryker Inc., Kalamazoo, Michigan, USA) is a lightweight porous form (150 – 400 µm) of PP. PP has the additional advantage over HA that the orbital implants do not need to be wrapped with autologous sclera/fascia or donor sclera/pericardium and that the muscles can be directly sutured onto the sphere, reducing both operating time and autologous donor site morbidity or immune response to allogenic donor tissue. They are also less costly to produce 2 . Moreover, a coupling device, or so-called “peg,” made of medical grade titanium, can be screwed directly into the porous implant and gives the surgeon and patient the option of coupling the implant to the prosthesis for further motility improvement 2 .

The most common complications of HA and PP are anterior implant surface exposures, occurring in 0.9 – 11% of enucleation and evisceration cases 2 , 4 , 9 , 10 , 11 . Some studies with lower exposure rates were conducted on patients with intraocular malignancies undergoing primary enucleation, while higher exposure rates were observed following secondary enucleations 2 , 3 .

The treatment of the smaller implant exposures includes application of topical antibiotic drops and simple observation or the use of an amniotic membrane or a vascularized conjunctival tissue flap to cover the defect 2 . Sometimes a revision of the socket is performed 12 . In rare cases of severe exposure associated with inflammation, the implants are removed 2 .

We report the unusual case of an acute PP (Medpor, Stryker Inc., Kalamazoo, Michigan, USA) orbital implant expulsion, without prior exposure, 14 months after primary enucleation for a choroidal melanoma, and speculate about the underlying mechanism(s).

## Case Report

A 61-year-old female presented with a sudden loss of vision in the left eye (LE). On examination, visual acuity was limited to hand movements. Indirect ophthalmoscopy, followed by B-scan ultrasonography, led to the diagnosis of a mushroom-shaped, inferior choroidal melanoma with a thickness of 7.5 mm and associated with a hemorrhagic and bullous secondary retinal detachment. General history was relevant for alcohol and substance abuse, as well as smoking. A systemic checkup did not reveal any metastases. Patient was treated with a primary enucleation of the LE.

Surgery was performed according to the usual procedure. Following a 360° limbal peritomy and disinsertion of the muscles, with the recti muscles being secured with 5.0 vicryl sutures, the optic nerve was sectioned with a snare wire, and the eyeball was removed. While using a PP glide, a 20-mm spherical PP implant (Medpor, Stryker Inc., Kalamazoo, Michigan, USA) was introduced deep into the orbit, and the four recti muscles were fixed onto it in a cross-shaped fashion. Tenonʼs capsule and the conjunctiva were closed with continuous 5.0 and 6.0 Vicryl sutures, respectively. A medium-sized, perforated, rigid conformer (FCI, Paris, France) with ofloxacin ointment (Floxal, Bausch & Lomb Swiss AG, Zug, Switzerland) and a compressive eye patch were then applied. After an uneventful night, the patient was discharged on a TID ofloxacin ointment topical treatment.

Ten days after the enucleation, the patient presented at the emergency department, complaining of periorbital pain, eyelid edema, yellow secretions, and loss of the conformer. With the presumed diagnosis of an orbital cellulitis, the patient was admitted and treated with intravenous (2.2 g TID for 3 days) and then oral (1 g BD for 5 days) co-amoxicillin (Sandoz Pharmaceuticals AG, Rotkreuz, Switzerland), all the while the ofloxacin ointment TID was continued. A conjunctival swab came back positive for “ *Staphylococcus aureus* (growth after enrichment),” sensitive to the administered antibiotics. Because of the swelling, oral prednisone was prescribed for about 10 days, at rapidly degressive doses (50 – 20 – 10 – 5 mg). After 4 days, the patient was discharged, with the TID ofloxacin ointment to be replaced by tobramycin/dexamethasone ointment (Tobradex Novartis Pharma Schweiz AG, Rotkreuz, Switzerland) after a week. Three weeks later, the patient complained of residual itching, and, suspecting an allergy, the topical treatment was switched to neomycin/polymyxin/B-dexamethasone ointment (Maxitrol Novartis Pharma Schweiz AG, Rotkreuz, Switzerland).

**Fig. 1:**
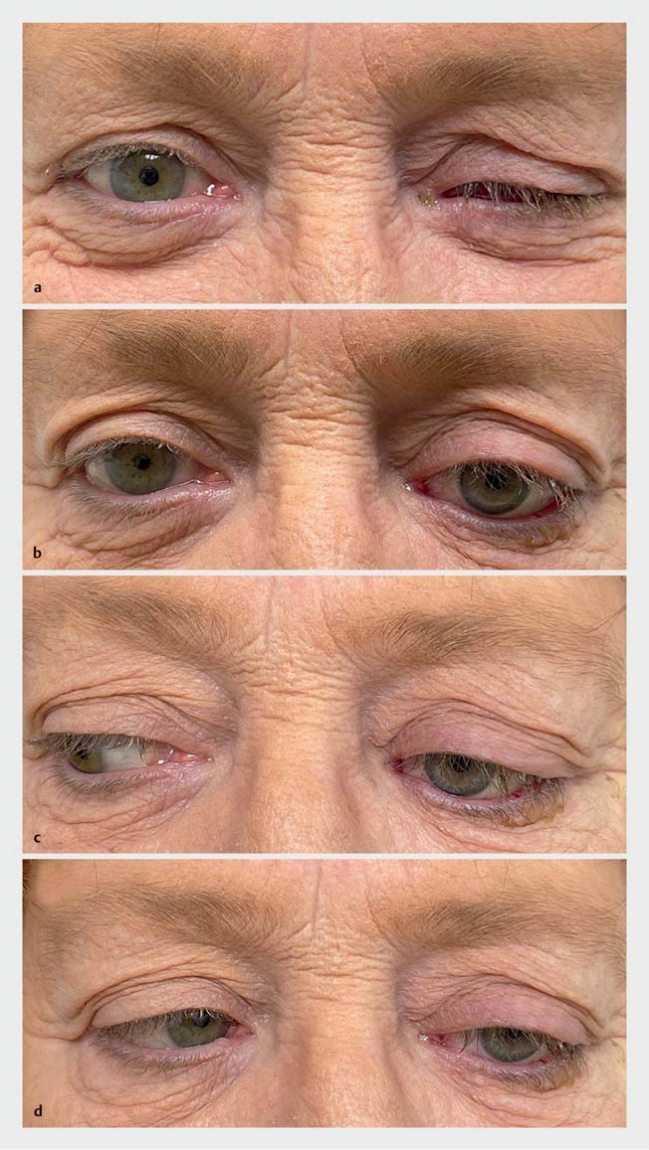
Adaptation of the artificial eye. **a** Without the ocular prosthesis. **b** – **d** With the prosthesis and reduced motility (with the patientʼs consent).

The swelling completely regressed, and a custom resin esthetic prosthesis was adapted 11 weeks following enucleation, with reduced motility ( Fig. 1 ). Because of persistent complaints of itching, with a slight conjunctival hyperemia and a whitish secretion, a new swab was performed, which turned out positive for “ *Staphylococcus aureus* (feeble quantity),” sensitive again to the applied antibiotics. Another topical treatment was prescribed, this time consisting of dexamethasone (Dexafree Théa Pharma S.A., Schaffhausen, Switzerland), ketotifen (Zaditen Omnivision AG, Neuhausen am Rheinfall, Switzerland) drops, and fusidic acid ointment (Fucithalmic Advanz Pharma Specialty Medicine Switzerland GmbH, Zürich, Switzerland). Orbital (MRI) resonance imaging was performed, of which the report excluded an abscess, and described a diffuse swelling of the anterior orbital soft tissues, but without involvement of the brain or bone. Upon advice of the infectious disease department, oral co-amoxicillin (Sandoz Pharmaceuticals AG, Rotkreuz, Switzerland) (1 g TID for 7 days) was reintroduced, and the patient was referred to an orbital specialist to evaluate the indication for orbital implant removal.

In the absence of fever or general deterioration, eyelid swelling, reduced motility, or periorbital pain, our colleagues opted for a conservative approach. The patient abandoned wearing her prothesis, as she was more comfortable without it. Several topical treatments were tried, including rinsing with just NaCl 0.9%, or cold black tea solutions. Artificial tears did not improve the continuing complaints of itching and whitish secretion either.

**Fig. 2:**
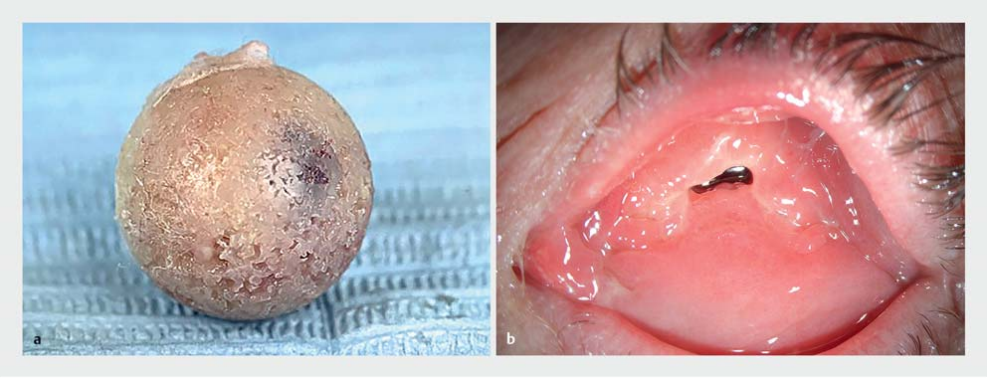
Implant expulsion from the orbital cavity. **a** The expulsed polyethylene implant, free from any fibrovascular tissue; **b** Conjunctival expulsion site.

Seven months later, during a multidisciplinary team discussion, it was hypothesized that the absence of the prothesis in the socket was sustaining the inflammation, and the patient was encouraged to resume its wear, associated with dexamethasone (Dexafree Théa Pharma S.A., Schaffhausen, Switzerland) and ketotifen (Zaditen Omnivision AG, Neuhausen am Reinfall, Switzerland) drops. The patient had also been observed constantly rubbing her anophthalmic socket during consultations and the possibility of a patient-related “mechanic” etiology was evoked. Finally, 14 months after enucleation, the PP implant was spontaneously expelled from the orbital socket, completely free from any fibrovascular tissue ( Fig. 2 ), following only a 2-day episode of increased conjunctival inflammation and rapidly progressive implant exposure. The microbiological analysis of the orbital implant revealed the presence of *Staphylococcus aureus, Prevotella denticola* , and *Penicillium* sp. and the conjunctival swab was positive again for “ *Staphylococcus aureus* (feeble quantity).” The patient was treated with systemic and topical antibiotics. A reconstruction with a dermis fat graft was proposed, which she has, so far, declined.

## Discussion

We describe the case history of a late and unusual acute PP spherical orbital implant expulsion and try to understand its underlying mechanisms.

First, a chronic low-grade inflammation had been present and possibly potentiated a conjunctival tissue breach, leading to the implant expulsion out of the socket 12 , 13 . It has been reported that orbital cellulitis is a risk factor for orbital implant extrusion for eyes undergoing evisceration for endophthalmitis 14 . Unusual in this case is that the first acute episode of presumed orbital cellulitis presented after only 10 days and not the usual 3 – 5 days. Also, once appropriate antibiotics have been administered, most cases resolve. One could speculate that some microbes survived in the porous lumina, where antibiotics could not reach them and/or that they created a biofilm preventing the ingrowth of fibrovascular tissue. Some surgeons therefore advocate for the implant “to have an antibiotic bath” before its introduction. Interestingly, in this patient, there was never a recurrence of swelling or fever. Her predominant continuing complaint was “itching” and a whitish secretion. Consequently, the repetitively positive, though feeble, *S. aureus* tests had been interpreted as normal skin contaminants, also because the patient had been observed to constantly rub her eye.

Second, since our patient was a heavy smoker, this may have disturbed the orbital socket microcirculation, which could have contributed to poor healing of the tissues covering the implant. Interestingly, in one rhinoplasty case series, smoking was significantly associated with PP implant extrusion 13 .

Third, gradual orbital tissue restitution has been reported to lead to implant extrusion after forced implantation, described as “the cactus syndrome” 12 . The authors suggest that using a PP glide for deep orbital implantation may help to avoid this phenomenon, which we did. Also, their case series mainly includes HA, and only one PP implant. According to Blaydon et al., the surface of the PP implant is much smoother than that of HA and causes less drag during placement in the soft orbital tissues 2 .

The fact that there was no ingrowth at all struck us. An MRI study found enhancement areas corresponding to the fibrovascular ingrowth in most patients at 1.5 months following enucleation 15 . Moreover, in our case, the PP orbital implant had been unwrapped, and it is believed that fibrovascular ingrowth is promoted by unwrapped porous PP implants 2 , 4 , 6 .

Alternatively, a fourth explanation is an orbital implant rejection, caused by an allergic type of immune response when local tissues fail to integrate with the alloplastic implant. However, this has been rarely reported in the literature 7 , 13 , and the PP material has been reported as “nonallergic”. Interestingly, sterile eosinophilic immune-mediated extrusion of a silicone orbital implant was described in a dog. This process suggests a major role of the Th2 immune response typically involved in allergies 7 . In our case, the patient suffered from persistent itching, unrelievable by antihistamine drops, thus, a chronic orbital inflammation could have come from a hypersensitivity response. The presence of *S. aureus* , which may only be an incidental finding, may also have triggered the immune response.

## Conclusion

In conclusion, our case represents a rare phenomenon of an acute and complete orbital implant expulsion, free from any fibrovascular tissue, more than 14 months following primary enucleation for uveal melanoma. We speculate about its precipitating factors, such as chronic low-grade *S. aureus* infection, smoking, cactus syndrome, and a potential PP allergy. Further studies are required to investigate the exact mechanisms of this type of PP spherical implant expulsion.
